# Supplementary material for: Linear ubiquitination by LUBEL has a role in Drosophila heat stress response
Source: EMBO Rep. 2016 Oct 4;17(11):1624–40. doi: 10.15252/embr.201642378 (PMC5090701; doi:10.15252/embr.201642378)
Supplement: Supplementary file 1 — Appendix [file EMBR-17-1624-s001.pdf]

Asaoka *et al*., Appendix

Table of Contents

Figure S1 - Interaction of Ub chains and LUBEL conserved domains.
Figure S2 - Histological analysis of muscle in LUBEL mutant flies.

Table S1-2; DE immune response genes from the ANOVA-type analysis.
Table S1 - Control (*w-* parental, LUBEL mutants; *CC/SS*, *delR2* and transposon insertion (*Mi-el*) lines)
Table S2 - Pricked (*w-* parental, LUBEL mutants; *CC/SS*, *delR2* and transposon insertion (*Mi-el*) lines)

Table S3-8; Statistically significantly enriched Gene Ontology terms within the DE genes of the individual pairwise comparisons.
Table S3 - control *CC/SS* mutant vs. control *w-* parental
Table S4 - control *delR2* mutant vs. control *w-* parental
Table S5 - control *Mi-el* mutant vs. control *w-* parental
Table S6 - pricked *CC/SS* mutant vs. pricked *w-* parental
Table S7 - pricked *delR2* mutant vs. pricked *w-* parental
Table S8 - pricked *Mi-el* mutant vs. pricked *w-* parental

Table S9 - Primers used to clone full length LUBEL.

Figure S1.

Ub chain interaction of LUBEL domains. Immobilized GST-NZF, GST-UBA2 , or GST-UBA1 was incubated with Lys (K) 48-, K 63-linked, or linear polyUb chains (2-7 ubiquitins) and bound Ub chains were subjected to immunoblotting using anti-Ub antibody or a linear linkage-specific Ub antibody (anti-linear Ub antibody). Loading of GST-proteins was visualized by Ponceau S staining.

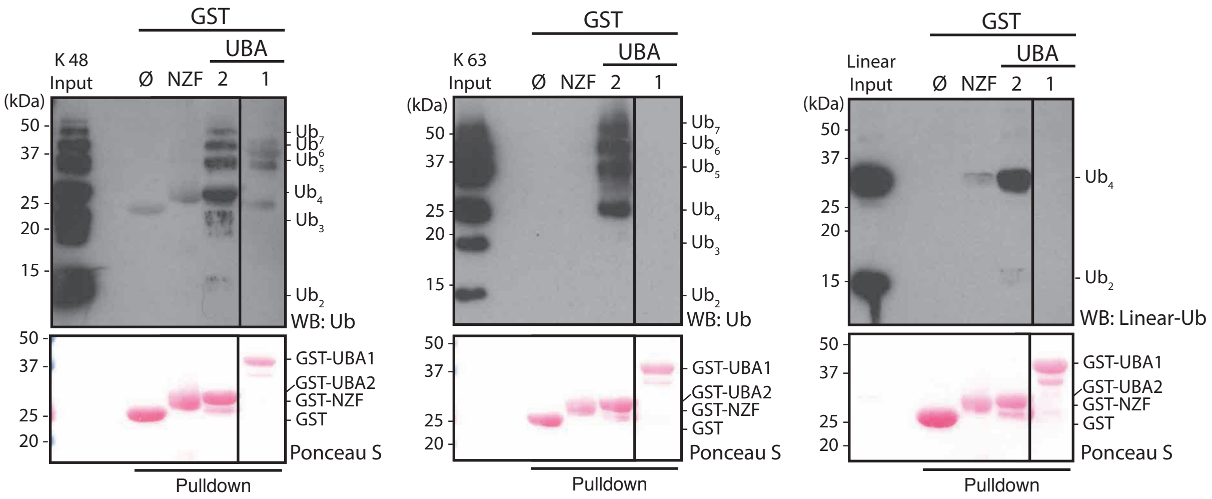

Figure S2.

A Histological analysis of muscle in *LUBEL* mutant flies. Hematoxylin and eosin (top panel) and actin (green) staining of thorax muscles from aged (Day 60) *delR2* female flies. Scale bars represent = 50 µm.

B Histological analysis of muscle in *LUBEL* mutant male flies. Hematoxylin and eosin (top panel) and actin (green) staining of thorax muscles from young (Day 3) or aged (Day 50) *w-* and *CC/SS*, and *delR2* (aged only) male flies. Scale bars represent = 50 µm.

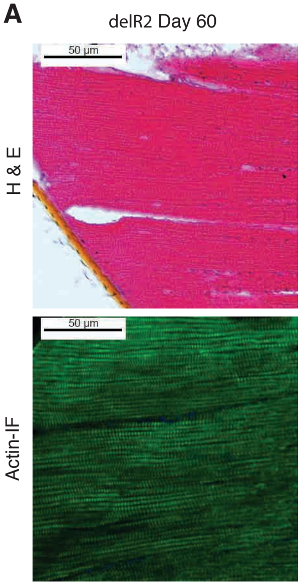

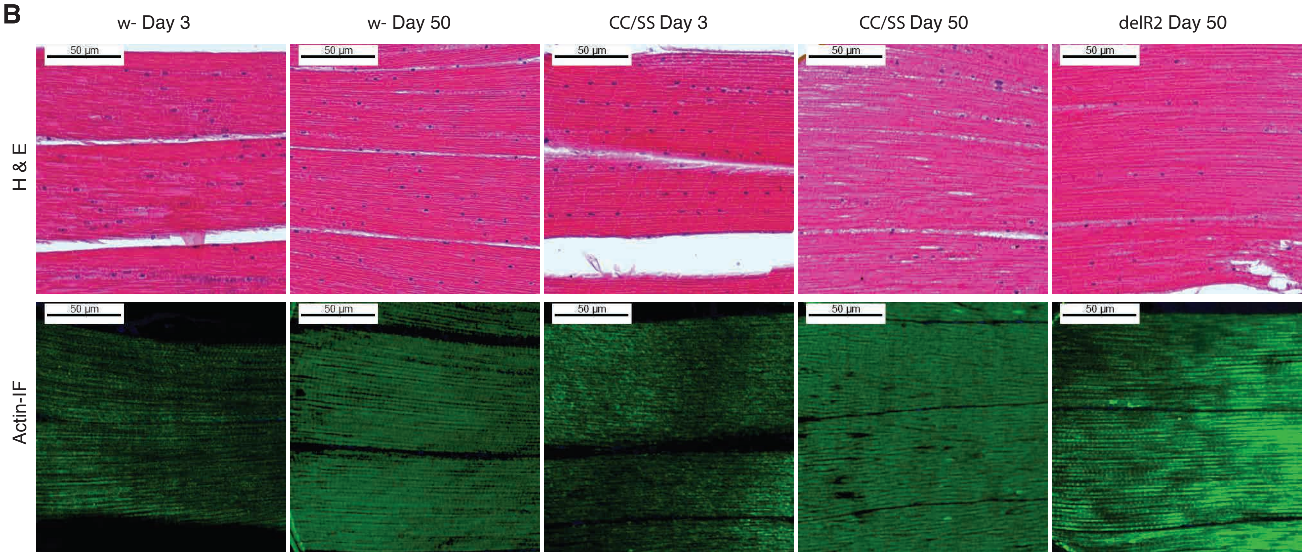

Table S1: DE (differentially expressed) immune response genes from the ANOVA-type analysis. These 11 out of a total of 402 immune response genes were statistically significantly DE between any of the four control fly lines.

Control (*w-* parental, LUBEL mutants; *CC/SS* , *delR2* and transposon insertion (*Mi-el*) lines)

	P.Value	adj.P.Val
LysD	9.71E-09	7.84E-06
LysC	3.14E-07	8.96E-05
LysB	1.10E-06	0.0002009
edin	2.76E-06	0.0003751
Sr-CI	7.54E-06	0.00081507
LysS	1.53E-05	0.00141953
wisp	2.67E-05	0.00210385
AttA	6.05E-05	0.00359838
CG11159	0.00034802	0.01259127
PGRP-SB1	0.00084538	0.02423955
LysE	0.00113544	0.03018644

DE genes filtered with FDR < 0.05 and logFC > 1.

Table S2: DE (differentially expressed) immune response genes from the ANOVA-type analysis. These 36 out of a total of 402 immune response genes were statistically significantly DE between any of the four pricked fly lines.

Pricked (*w-* parental, LUBEL mutants; *CC/SS* , *delR2* and transposon insertion (*Mi-el*) lines)

	P.Value	adj.P.Val
LysD	1.05E-08	1.70E-06
Nplp2	6.22E-08	5.52E-06
AttA	8.77E-08	6.72E-06
eater	3.19E-07	1.67E-05
PGRP-SC1a	5.86E-07	2.51E-05
LysB	7.69E-07	3.11E-05
LysP	9.39E-07	3.58E-05
PGRP-SD	9.59E-07	3.65E-05
Tep1	1.20E-06	4.35E-05
PGRP-SC2	1.60E-06	5.27E-05
edin	1.63E-06	5.35E-05
LysC	1.76E-06	5.65E-05
spirit	1.93E-06	6.03E-05
pirk	2.08E-06	6.35E-05
fon	2.69E-06	7.57E-05
Hml	5.08E-06	0.00012144
Sr-CI	5.38E-06	0.0001266
PGRP-SB1	1.18E-05	0.00022415
PGRP-SA	1.81E-05	0.00030366
Tep2	3.07E-05	0.00044343
CecC	3.22E-05	0.00045772
LysS	7.60E-05	0.00082594
PGRP-SC1b	0.00012057	0.00115962
CecB	0.0001274	0.00120417
wisp	0.00014483	0.00132088
IM23	0.00016661	0.00144474
Toll-9	0.0003279	0.0023726
AttD	0.00059408	0.00363418
Cpr49Ac	0.00085878	0.00467034
LysX	0.0013008	0.00626509
CG11159	0.00143156	0.00674451
proPO-A1	0.00205867	0.00885833
Def	0.00334494	0.01270714
Spn28Dc	0.00623615	0.0203928
PGRP-SB2	0.01312451	0.03599034
Bro	0.01663429	0.04317598

DE genes filtered with FDR < 0.05 and logFC > 1.

Table S3: Statistically significantly enriched Gene Ontology terms within the DE genes of the individual pairwise comparisons.

control *CC/SS* mutant vs. control *w-* parental

Gene Ontology ID	Term	P value
GO:0010171	body morphogenesis	0.00019
GO:0018126	protein hydroxylation	0.00038
GO:0018401	peptidyl-proline hydroxylation to 4-hydroxy-L-pr	0.00038
GO:0019471	4-hydroxyproline metabolic process	0.00038
GO:0019511	peptidyl-proline hydroxylation	0.00038
GO:0018208	peptidyl-proline modification	0.00061
GO:0040003	chitin-based cuticle development	0.00119
GO:1901605	alpha-amino acid metabolic process	0.0017
GO:0055114	oxidation-reduction process	0.00348
GO:0042335	cuticle development	0.00562
GO:0006575	cellular modified amino acid metabolic process	0.00655
GO:0006022	aminoglycan metabolic process	0.0354
GO:0018193	peptidyl-amino acid modification	0.0428
GO:0006520	cellular amino acid metabolic process	0.0429

Table S4: Statistically significantly enriched Gene Ontology terms within the DE genes of the individual pairwise comparisons.

control *delR2* mutant vs. control *w-* parental

Gene Ontology ID	Term	P value
GO:0010171	body morphogenesis	0.00023
GO:0018126	protein hydroxylation	0.00046
GO:0018401	peptidyl-proline hydroxylation to 4-hydroxy-	0.00046
GO:0019471	4-hydroxyproline metabolic process	0.00046
GO:0019511	peptidyl-proline hydroxylation	0.00046
GO:0018208	peptidyl-proline modification	0.00078
GO:1901605	alpha-amino acid metabolic process	0.00225
GO:0006575	cellular modified amino acid metabolic proce	0.00817
GO:0040003	chitin-based cuticle development	0.0087
GO:0007606	sensory perception of chemical stimulus	0.0139
GO:0055114	oxidation-reduction process	0.0152
GO:0042335	cuticle development	0.0294
GO:0006022	aminoglycan metabolic process	0.043
GO:0006508	proteolysis	0.0494

Table S5: Statistically significantly enriched Gene Ontology terms within the DE genes of the individual pairwise comparisons.

control *Mi-el* mutant vs. control *w-* parental

Gene Ontology ID	Term	P value
GO:0017085	response to insecticide	1.40E-05
GO:0009636	response to toxic substance	7.30E-05
GO:0016998	cell wall macromolecule catabolic process	0.00012
GO:0044036	cell wall macromolecule metabolic process	0.00012
GO:0071554	cell wall organization or biogenesis	0.00012
GO:0006805	xenobiotic metabolic process	0.00038
GO:0009410	response to xenobiotic stimulus	0.00038
GO:0071466	cellular response to xenobiotic stimulus	0.00038
GO:0009631	cold acclimation	0.00116
GO:0014074	response to purine-containing compound	0.0017
GO:0031000	response to caffeine	0.0017
GO:0009404	toxin metabolic process	0.00238
GO:0017143	insecticide metabolic process	0.00238
GO:0019730	antimicrobial humoral response	0.00265
GO:0009409	response to cold	0.00418
GO:0006959	humoral immune response	0.00654
GO:0043279	response to alkaloid	0.00679
GO:0007606	sensory perception of chemical stimulus	0.00758
GO:0010955	negative regulation of protein processing	0.00774
GO:1903318	negative regulation of protein maturation	0.00774
GO:0043207	response to external biotic stimulus	0.0102
GO:0051707	response to other organism	0.0102
GO:0007600	sensory perception	0.0108
GO:0009607	response to biotic stimulus	0.0108
GO:0050909	sensory perception of taste	0.0187
GO:0030010	establishment of cell polarity	0.0205
GO:0009593	detection of chemical stimulus	0.0264
GO:0070613	regulation of protein processing	0.0265
GO:1903317	regulation of protein maturation	0.0265
GO:0006508	proteolysis	0.0293
GO:0032507	maintenance of protein location in cell	0.0335
GO:0010951	negative regulation of endopeptidase activity	0.0368
GO:0045861	negative regulation of proteolysis	0.0368
GO:0010466	negative regulation of peptidase activity	0.0406
GO:0009266	response to temperature stimulus	0.041
GO:0045185	maintenance of protein location	0.0415
GO:0051651	maintenance of location in cell	0.0474
GO:0055114	oxidation-reduction process	0.0486

Table S6: Statistically significantly enriched Gene Ontology terms within the DE genes of the individual pairwise comparisons.

pricked *CC/SS* mutant vs. pricked *w-* parental

Gene Ontology ID	Term	P value
GO:0040003	chitin-based cuticle development	5.60E-14
GO:0042335	cuticle development	1.70E-13
GO:0006022	aminoglycan metabolic process	6.40E-09
GO:0006030	chitin metabolic process	1.60E-08
GO:1901071	glucosamine-containing compound metabolic pr	4.00E-08
GO:0006040	amino sugar metabolic process	4.60E-08
GO:0055114	oxidation-reduction process	3.10E-05
GO:0018401	peptidyl-proline hydroxylation to 4-hydroxy-L-pr	4.50E-05
GO:0019471	4-hydroxyproline metabolic process	4.50E-05
GO:0019511	peptidyl-proline hydroxylation	4.50E-05
GO:0018126	protein hydroxylation	6.40E-05
GO:0006035	cuticle chitin biosynthetic process	0.00023
GO:1901605	alpha-amino acid metabolic process	0.00036
GO:0006031	chitin biosynthetic process	0.00044
GO:1901073	glucosamine-containing compound biosynthetic	0.00044
GO:0046349	amino sugar biosynthetic process	0.00076
GO:0018208	peptidyl-proline modification	0.00091
GO:0010038	response to metal ion	0.00091
GO:0009617	response to bacterium	0.00105
GO:0006023	aminoglycan biosynthetic process	0.00119
GO:0009620	response to fungus	0.00159
GO:0006575	cellular modified amino acid metabolic process	0.0016
GO:0006034	cuticle chitin metabolic process	0.00174
GO:0034644	cellular response to UV	0.00174
GO:0008202	steroid metabolic process	0.00192
GO:0043207	response to external biotic stimulus	0.0025
GO:0051707	response to other organism	0.0025
GO:0044550	secondary metabolite biosynthetic process	0.00267
GO:0009607	response to biotic stimulus	0.00268
GO:0050832	defense response to fungus	0.00295
GO:0007606	sensory perception of chemical stimulus	0.004
GO:0050830	defense response to Gram-positive bacterium	0.00492
GO:0006026	aminoglycan catabolic process	0.00527
GO:0042438	melanin biosynthetic process	0.00544
GO:0006508	proteolysis	0.00567
GO:0098542	defense response to other organism	0.0089
GO:0010035	response to inorganic substance	0.00995

GO:1901136	carbohydrate derivative catabolic process	0.0109
GO:0006032	chitin catabolic process	0.0119
GO:0019748	secondary metabolic process	0.0125
GO:0010171	body morphogenesis	0.014
GO:0034605	cellular response to heat	0.014
GO:0046348	amino sugar catabolic process	0.014
GO:1901072	glucosamine-containing compound catabolic pro	0.014
GO:0006520	cellular amino acid metabolic process	0.0158
GO:0006081	cellular aldehyde metabolic process	0.0162
GO:0046189	phenol-containing compound biosynthetic proce	0.0162
GO:0042180	cellular ketone metabolic process	0.0177
GO:0045455	ecdysteroid metabolic process	0.0192
GO:0032787	monocarboxylic acid metabolic process	0.0205
GO:0060439	trachea morphogenesis	0.0242
GO:0042445	hormone metabolic process	0.0269
GO:1901565	organonitrogen compound catabolic process	0.0274
GO:0006952	defense response	0.0289
GO:1901135	carbohydrate derivative metabolic process	0.0299
GO:0046692	sperm competition	0.0305
GO:0009411	response to UV	0.034
GO:0034754	cellular hormone metabolic process	0.0344
GO:1901617	organic hydroxy compound biosynthetic process	0.0344
GO:0007320	insemination	0.0376
GO:0048067	cuticle pigmentation	0.0376
GO:0019731	antibacterial humoral response	0.0455
GO:0050909	sensory perception of taste	0.0486
GO:0042742	defense response to bacterium	0.0488

Table S7: Statistically significantly enriched Gene Ontology terms within the DE genes of the individual pairwise comparisons.

pricked *delR2* mutant vs. pricked *w-* parental

Gene Ontology ID	Term	P value
GO:0006022	aminoglycan metabolic process	3.80E-07
GO:0006030	chitin metabolic process	3.90E-07
GO:1901071	glucosamine-containing compound metabolic pr	7.60E-07
GO:0006040	amino sugar metabolic process	8.30E-07
GO:0040003	chitin-based cuticle development	9.70E-05
GO:0010171	body morphogenesis	0.00021
GO:0042335	cuticle development	0.001
GO:0018401	peptidyl-proline hydroxylation to 4-hydroxy-L-pr	0.00193
GO:0019471	4-hydroxyproline metabolic process	0.00193
GO:0019511	peptidyl-proline hydroxylation	0.00193
GO:0006575	cellular modified amino acid metabolic process	0.002
GO:0018126	protein hydroxylation	0.00234
GO:0018208	peptidyl-proline modification	0.00469
GO:1901135	carbohydrate derivative metabolic process	0.0113
GO:0007606	sensory perception of chemical stimulus	0.0123
GO:0006508	proteolysis	0.0156
GO:0009620	response to fungus	0.0192
GO:1901605	alpha-amino acid metabolic process	0.0224
GO:1901136	carbohydrate derivative catabolic process	0.0462

Table S8: Statistically significantly enriched Gene Ontology terms within the DE genes of the individual pairwise comparisons.

pricked *Mi-el* mutant vs. pricked *w-* parental

Gene Ontology ID	Term	P value
GO:0040003	chitin-based cuticle development	5.40E-09
GO:0006022	aminoglycan metabolic process	2.00E-08
GO:0042335	cuticle development	2.80E-08
GO:0006030	chitin metabolic process	9.40E-08
GO:1901071	glucosamine-containing compound metabolic process	2.70E-07
GO:0006040	amino sugar metabolic process	3.10E-07
GO:0017085	response to insecticide	1.90E-06
GO:0006805	xenobiotic metabolic process	2.90E-05
GO:0009410	response to xenobiotic stimulus	2.90E-05
GO:0071466	cellular response to xenobiotic stimulus	2.90E-05
GO:0009636	response to toxic substance	8.20E-05
GO:0055114	oxidation-reduction process	0.00013
GO:0006508	proteolysis	2.00E-04
GO:0009404	toxin metabolic process	0.00069
GO:0017143	insecticide metabolic process	0.00069
GO:0006026	aminoglycan catabolic process	0.00069
GO:0006035	cuticle chitin biosynthetic process	0.00085
GO:0006031	chitin biosynthetic process	0.00164
GO:1901073	glucosamine-containing compound biosynthetic process	0.00164
GO:0046349	amino sugar biosynthetic process	0.00278
GO:0006959	humoral immune response	0.00333
GO:0000270	peptidoglycan metabolic process	0.0043
GO:0006027	glycosaminoglycan catabolic process	0.0043
GO:0009253	peptidoglycan catabolic process	0.0043
GO:1901136	carbohydrate derivative catabolic process	0.00476
GO:0006034	cuticle chitin metabolic process	0.00624
GO:0007586	digestion	0.00862
GO:0014074	response to purine-containing compound	0.00862
GO:0031000	response to caffeine	0.00862
GO:0060439	trachea morphogenesis	0.0156
GO:0008202	steroid metabolic process	0.0165
GO:0045455	ecdysteroid metabolic process	0.0217
GO:0003383	apical constriction	0.0276
GO:0001101	response to acid chemical	0.0314
GO:0007606	sensory perception of chemical stimulus	0.0363
GO:0006955	immune response	0.0386
GO:0006023	aminoglycan biosynthetic process	0.0387
GO:0006032	chitin catabolic process	0.0387

GO:0010951	negative regulation of endopeptidase activity	0.0387
GO:0019748	secondary metabolic process	0.042
GO:0034754	cellular hormone metabolic process	0.0429
GO:0043279	response to alkaloid	0.0442
GO:0060438	trachea development	0.0442
GO:0010466	negative regulation of peptidase activity	0.0449
GO:0046348	amino sugar catabolic process	0.0449
GO:0070252	actin-mediated cell contraction	0.0449
GO:1901072	glucosamine-containing compound catabolic process	0.0449
GO:0042445	hormone metabolic process	0.0489
GO:0019730	antimicrobial humoral response	0.0495

Table S9.
Primers used to amplify and clone full length LUBEL. Five fragments were amplified to clone Dmel_CG11321, isoform E in pAMW insect expression vector by Gibson assembly. Fragments 1 to 4 were amplified from cDNA of male adult fly, while fragment 5 was amplified from pGEX6p1-LUBEL-RBR-C.

1st fragment
Fwd 5’-TTCTGAAGAGGACTTGAATCAATGACGACCCACCAGTTGCTAAA-3‘
Rev 5’-TCAGACTCCAGATCGGAGTAAATGCGACTGGTTCTGGCCGAG-3’

2nd fragment
Fwd 5‘-GAACCAGTCGCATTTACTCCGATCTGGAGTCTGAGGGATCGGG-3’
Rev 5’-GGTTCTGCTTGGCCTGGTTCTGTTCCATGTTGCCAATGAG-3‘

3rd fragment
Fwd 5’-CAACA TGGAACAGAACCAGGCCAAGCAGAACCAGGAGG-3‘
Rev 5’-CGGACAACTGTTCGGATTTTGGAGAACTGACTGTTTCCCCTATGGG-3’

4th fragment
Fwd 5’-GGGAAACAGTCAGTTCTCCAAAATCCGAACAGT TGTCCG-3‘
Rev 5’-GGGATAGCTGTTCATGCAGAGCTCGCACTCCTGC-3’

5th fragment
Fwd 5’-CAGGAGTGCGAGCTCTGCATGAACAGCTATCCCATGAATC-3’
Rev 5’-GGTTCCTTCACAAAGATCCTGTCATGCCGATTTCAGTGGTATAT-3’
