# Supplementary material for: Linear ubiquitination by LUBEL has a role in Drosophila heat stress response
Source: EMBO Rep. 2016 Oct 4;17(11):1624–40. doi: 10.15252/embr.201642378 (PMC5090701; doi:10.15252/embr.201642378)
Supplement: Supplementary file 2 — Expanded View Figures PDF [file EMBR-17-1624-s002.pdf]

Expanded View Figures

Figure EV1. Linkage types of Ub chains and predicted domains in CG11321.

A Schematics of eight different linkage types of Ub chains. Ub can form different types of polymers by conjugating via intrinsic Lys residues (K 6, K 11, K 27, K 29, K 33, K 48, and K 63) and M 1 (linear).

B A multiple amino acid sequence alignment of RBR-C in HOIP family members. Conserved residues are colored according to the ClustalX coloring scheme. Long unaligned regions in *A. mellifera* and *P. humanus* were replaced by the number of deleted residues in squared brackets. R1, IBR, R2, and LDD domains are indicated with gray bars above the sequences. The zinc (Zn)-coordinating residues known in *H. sapiens* HOIP-RBR-C are labeled below. A black triangle indicates the Cys (C) residue for the thioester intermediate, two * are Zn-coordinating Cys residues in R2 targeted to create catalytically dead mutants, and two O are residues mutated in LDD.

C–F Multiple amino acid sequence alignments of N-terminal HOIP domains, UBA1 (C), UBA2 (D), B-box (E), and NZF (F).

© 2016 The Authors

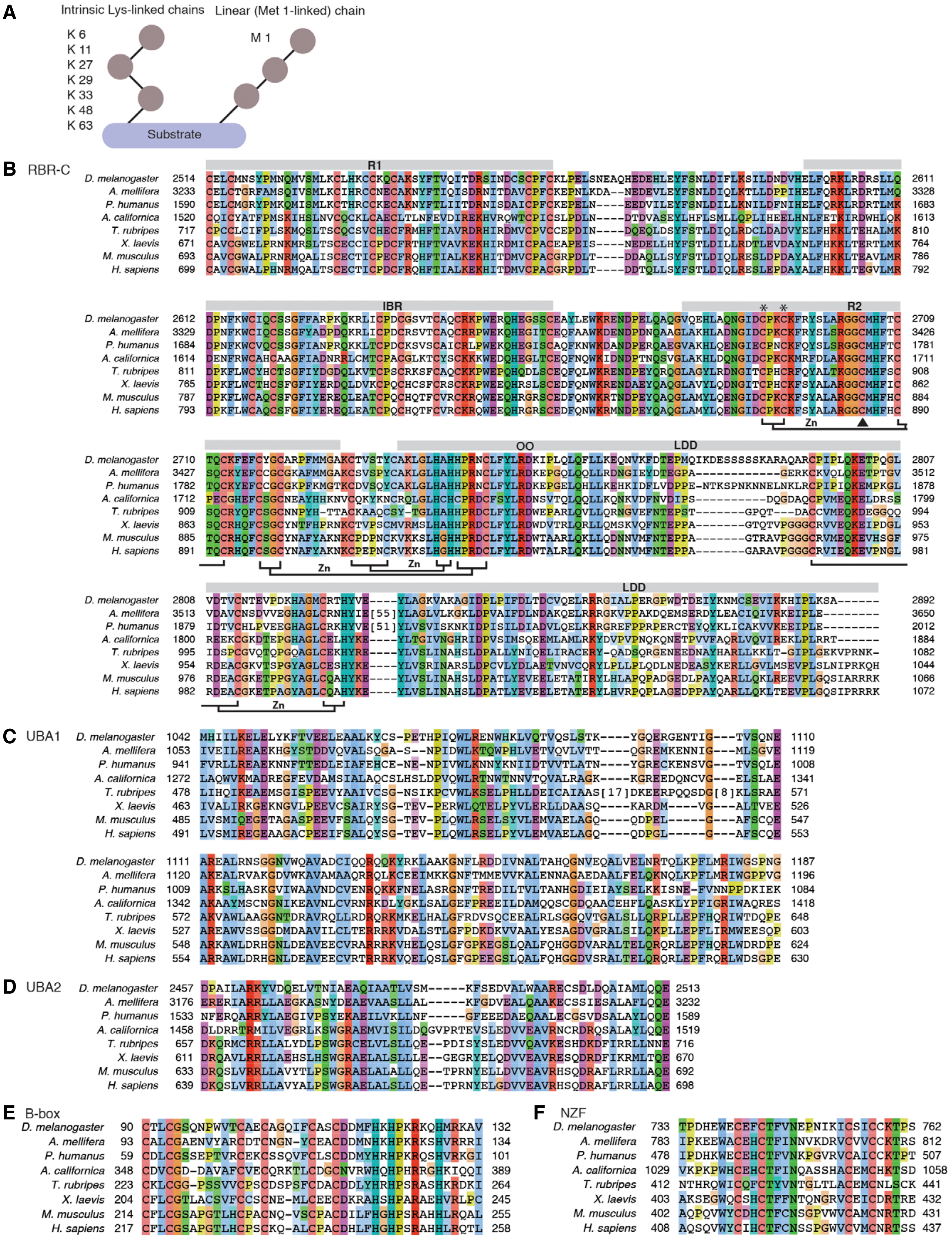
A
Intrinsic Lys-linked chains
Linear (Met 1-linked) chain
K 6
K 11
K 27
K 29
K 33
K 48
K 63
M 1
Substrate
B RBR-C
R1
IBR
R2
Zn
OO
LDD
C UBA1
D UBA2
E B-box
F NZF
D. melanogaster
A. mellifera
P. humanus
A. californica
T. rubripes
X. laevis
M. musculus
H. sapiens

Figure EV1.

Figure EV2. LUBEL-RBR-C specifically synthesizes linear Ub chains.

A *In vitro* ubiquitination assay of predicted LUBEL-RBR-C in combination with Ube1 and two different *Drosophila* E2s, UbcD10 and Effete/UbcD1, using nontagged or N-terminally His_6-tagged Ub. Synthesized Ub chains were analyzed by immunoblotting using anti-linear Ub antibody or anti-Ub antibody. Protein loading was visualized by Ponceau S staining. *: nonspecific band.

B Mass spectrometry analysis of the Ub chains generated by LUBEL-RBR-C and UbcD10. MS/MS spectra acquired from the linear Ub chain peptide using an identical sample as Fig 2A lane 4 is shown.

C, D *In vitro* deubiquitinating activities of vOTU (C), or OTULIN (D). Recombinant vOTU, OTULIN (WT or a catalytically dead C129A mutant) was incubated with K 48-, K 63-linked, or linear Ub_2 chains for indicated times. Subsequently, proteins were resolved on SDS–PAGE gels and stained with Coomassie dye. *: nonspecific band.

E *In vitro* ubiquitination assay of LUBEL-RBR-C C2690S/C2693S (CC/SS) mutant compared to WT. Linear Ub chain formation was analyzed by immunoblotting using anti-linear Ub antibody. Total amount of proteins was analyzed by Ponceau S staining. *: nonspecific band.

F Linear Ub chains in *Drosophila* S2 cells transfected with LUBEL-RBR-C. Myc-RBR-C WT or Myc-RBR-C CC/SS was transfected in S2 cells. Linear Ub chains in TCL were visualized by immunoblotting using anti-linear Ub antibody. Expression of RBR-C (WT or C2690/2693S) was analyzed by using anti-Myc-antibody, and tubulin was blotted to examine protein loading.

G *In vitro* ubiquitination assay using full-length LUBEL. *In vitro* ubiquitination assay was performed using recombinant full-length LUBEL purified by a baculovirus-based insect expression method, in combination with Ube1 and UbcD10. Amount of E3 ligase was determined by using an antibody raised against LUBEL-RBR-C. LUBEL-RBR-C was used as positive control.

H Catalytic activity of LUBEL-RBR-C with extended N-terminal UBA2 *in vitro*. *In vitro* ubiquitination assay was performed using LUBEL-RBR-C or LUBEL-UBA2-RBR-C in combination with Ube1 and UbcD10. Linear Ub chain formation was analyzed as (E), and amount of E3 ligase was determined by anti-RBR-C antibody. *: nonspecific band.

 © 2016 The Authors

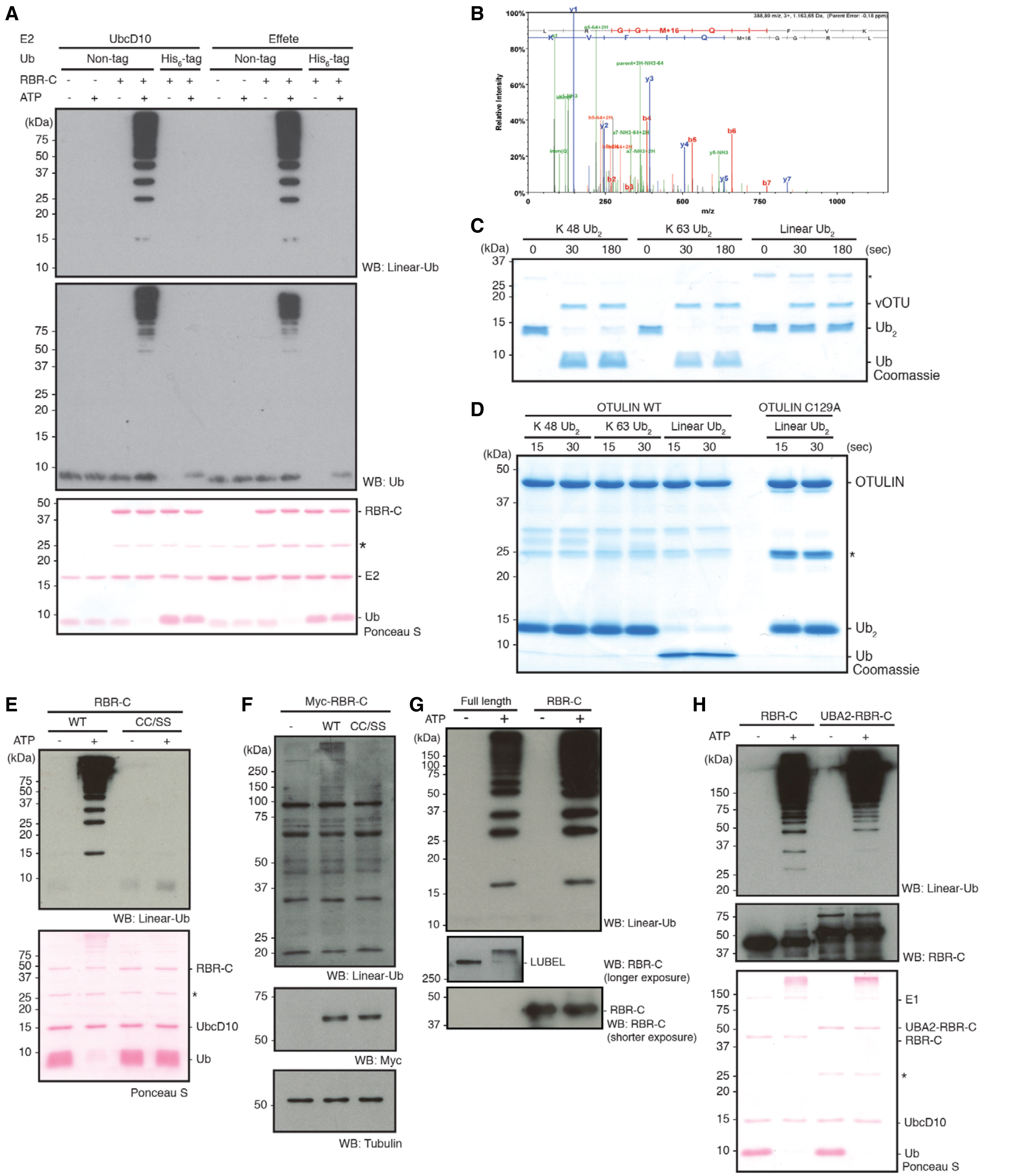
A
E2
UbcD10
Effete
Ub
Non-tag
His6-tag
RBR-C
ATP
(kDa)
WB: Linear-Ub
WB: Ub
RBR-C
E2
Ub
Ponceau S
B
Relative Intensity
m/z
C
K 48 Ub2
K 63 Ub2
Linear Ub2
(sec)
vOTU
Ub2
Ub
Coomassie
D
OTULIN WT
OTULIN C129A
OTULIN
E
RBR-C
WT
CC/SS
UbcD10
F
Myc-RBR-C
WB: Myc
WB: Tubulin
G
Full length
LUBEL
WB: RBR-C (longer exposure)
WB: RBR-C (shorter exposure)
H
UBA2-RBR-C
WB: RBR-C
E1

Figure EV2.

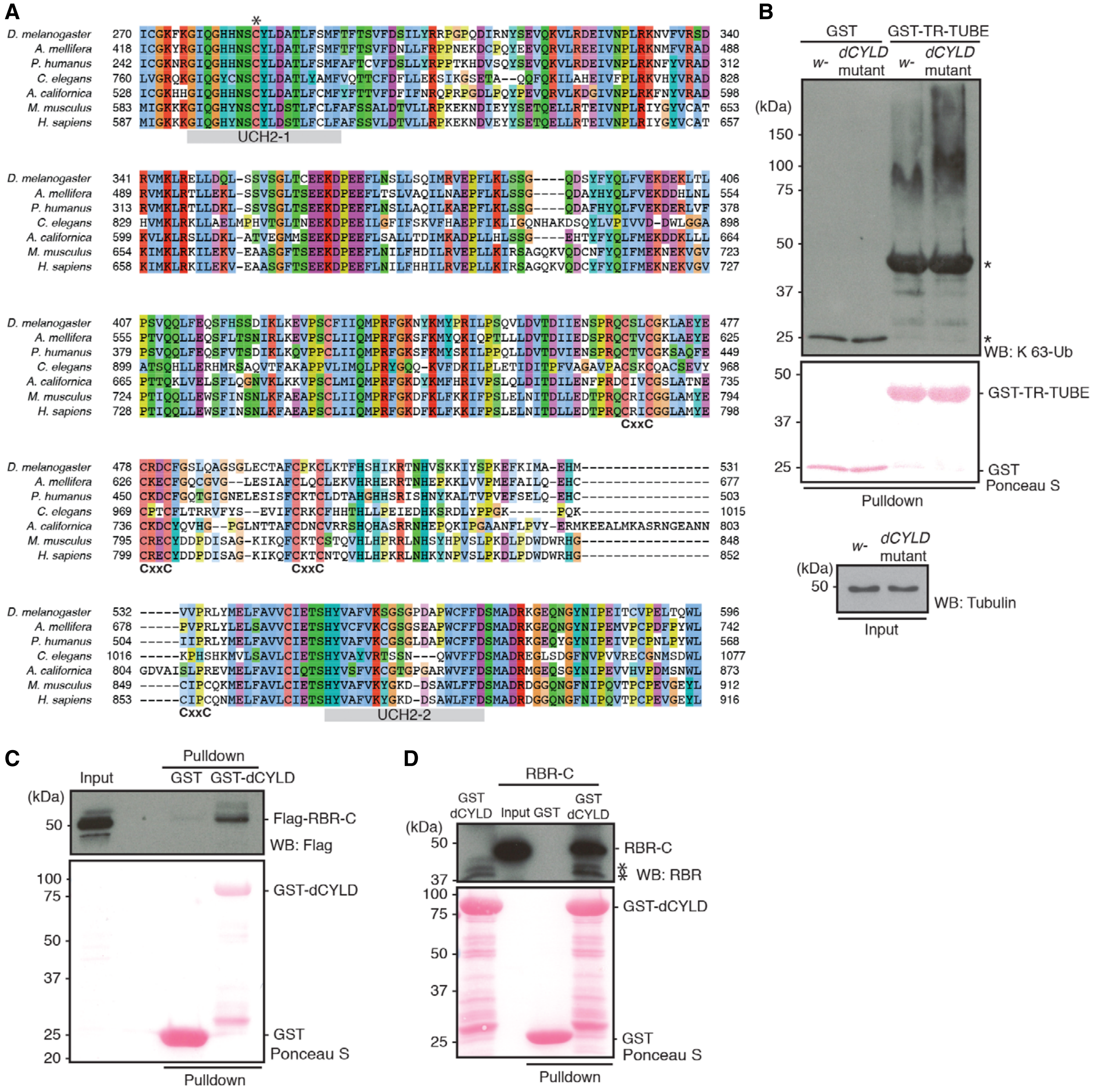

Figure EV3. Characteristics of *Drosophila* CYLD.

- A A multiple amino acid sequence alignment of the CYLD catalytic domain in different species. * indicates predicted catalytic Cys residue, while C-X-X-C pairs (labeled CxxC) and the UCH catalytic domains, UCH2-1 and UCH2-2 (in gray), are shown below the sequences.
- B Endogenous level of K 63-linked Ub chains in *dCYLD* mutant flies. Poly-Ub chains in the total protein extracts of w^{-} and *dCYLD* mutant were enriched by GST-TR-TUBE pulldown, and the samples were resolved and detected using anti-K 63-linked Ub chains. GST was used as control for the pulldown. Input of GST proteins was visualized by Ponceau S, and total protein extracts were blotted with anti-tubulin antibody. *: nonspecific band.
- C Interaction between dCYLD and LUBEL-RBR-C. Flag-RBR-C was transfected into S2 cells and total cell lysate was incubated with either agarose-immobilized GST or GST-dCYLD. After GST pulldown, samples were analyzed by immunoblotting using anti-Flag antibody. Loading of GST proteins was visualized by Ponceau S staining.
- D Protein–protein interaction of recombinant dCYLD and recombinant LUBEL-RBR-C. LUBEL-RBR-C purified from *E. coli* was incubated with immobilized GST or GST-dCYLD for pulldown assay. The interaction was analyzed by immunoblotting using anti-LUBEL-RBR antibody. Loading of GST proteins was visualized by Ponceau S staining. *: nonspecific band.

 © 2016 The Authors

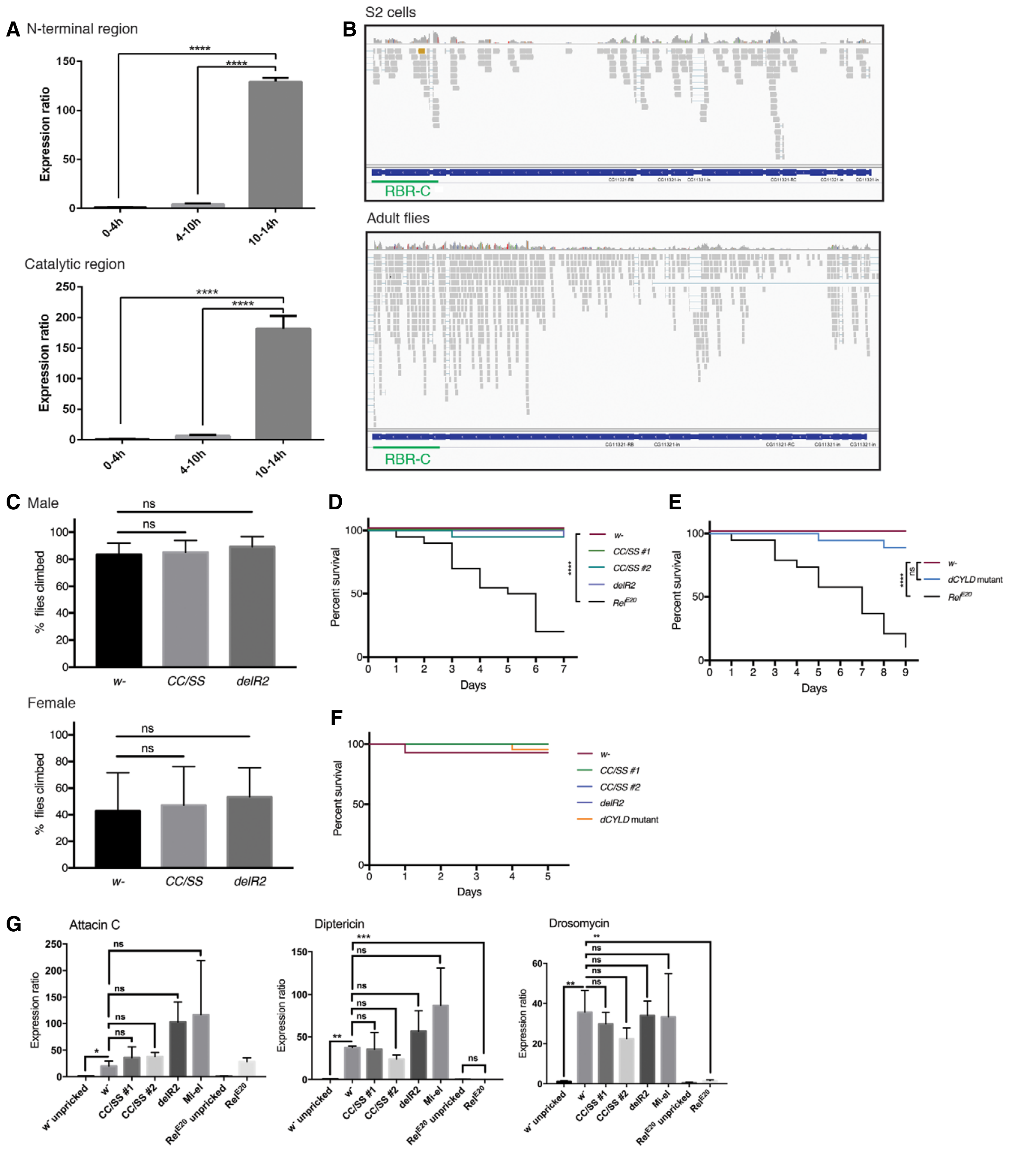
A N-terminal region
Expression ratio
0-4h
4-10h
10-14h
Catalytic region
B S2 cells
RBR-C
Adult flies
C Male
ns
% flies climbed
w-
CC/SS
delR2
Female
D
Percent survival
Days
w-
CC/SS #1
CC/SS #2
delR2
RelE20
E
dCYLD mutant
F
G Attacin C
Diptericin
Drosomycin
w- unpricked
w-
CC/SS #1
CC/SS #2
delR2
Mi-el
RelE20 unpricked
RelE20

Figure EV4.

Figure EV4. Establishing LUBEL mutant fly strains.

A Endogenous mRNA expression of LUBEL detected in different embryonic stages of ω^{-} flies by qPCR. RNA was isolated from embryos and the LUBEL expression levels were measured by qPCR. TATA binding protein (TBP) was used as a reference, and two sets of primers, N-terminal region or catalytic region, were used to detect all isoforms or only the long isoforms, respectively. Representative data are shown from three independent experiments.

B Endogenous mRNA fragments of LUBEL detected in S2 cells (above) and ω^{-} male adult flies (bottom) by RNA-Seq. The aligned transcripts were visualized using The Integrative Genomics Viewer (IGV_2.3.40 software) and screenshots of the CG11321 region are shown. Representative alignments of three repeats are shown.

C Negative geotaxis assay of aged male (top panel) and female (bottom panel) flies. The assays were repeated five times for each group (between 47 to 55 flies per group), and the results were combined to create the graphs. *t*-test analysis showed no significant difference between the lines. Representative videos can be found in Movies EV2 and EV3.

D, E Survival of LUBEL mutant and *dCYLD* mutant flies upon Gram-negative bacterial infection. Septic injury was performed using *E. coli* in 20 young adult male flies. Recovered flies were kept in 25°C and counted every 24 h until indicated time. Rel^{E20} was used as positive control for the assay. Curve comparison tests indicated there is no statistical difference except for Rel^{E20} > 0.0001. Representative survival curve is shown for the LUBEL mutant flies, *CC/SS* #1 and #2, and *delR2* (D; from four independent experiments), and catalytically dead dCYLD flies (E; from three independent experiments).

F Survival of adult male LUBEL mutant and *dCLYD* mutant flies by septic injury with Gram-positive *M. luteus* bacteria. Septic injury with *M. luteus* was performed and recovered flies were kept in 29°C and counted every 24 h for indicated time. Curve comparison tests indicated the difference is not significant between the lines. Representative data are shown from three independent experiments.

G Antimicrobial peptide induction upon septic injury in *LUBEL* mutant flies. Septic injury with *E. coli* was performed, and after 10-h recovery, RNA was isolated and mRNA levels of *Attacin C*, *Diptericin*, and *Drosomycin* were measured by qPCR. *Rp49* was used as a reference, and ω^{-} unpricked sample was used as calibrator to calculate the expression ratio. Multiple comparisons were performed using one-way ANOVA. Representative data are shown from three independent experiments.

Data information: Data in (A, C, G) are presented as mean ± SD. *$P < 0.05$, **$P < 0.005$, ***$P < 0.001$, ****$P < 0.0001$. Data were analyzed using unpaired *t*-test.

© 2016 The Authors

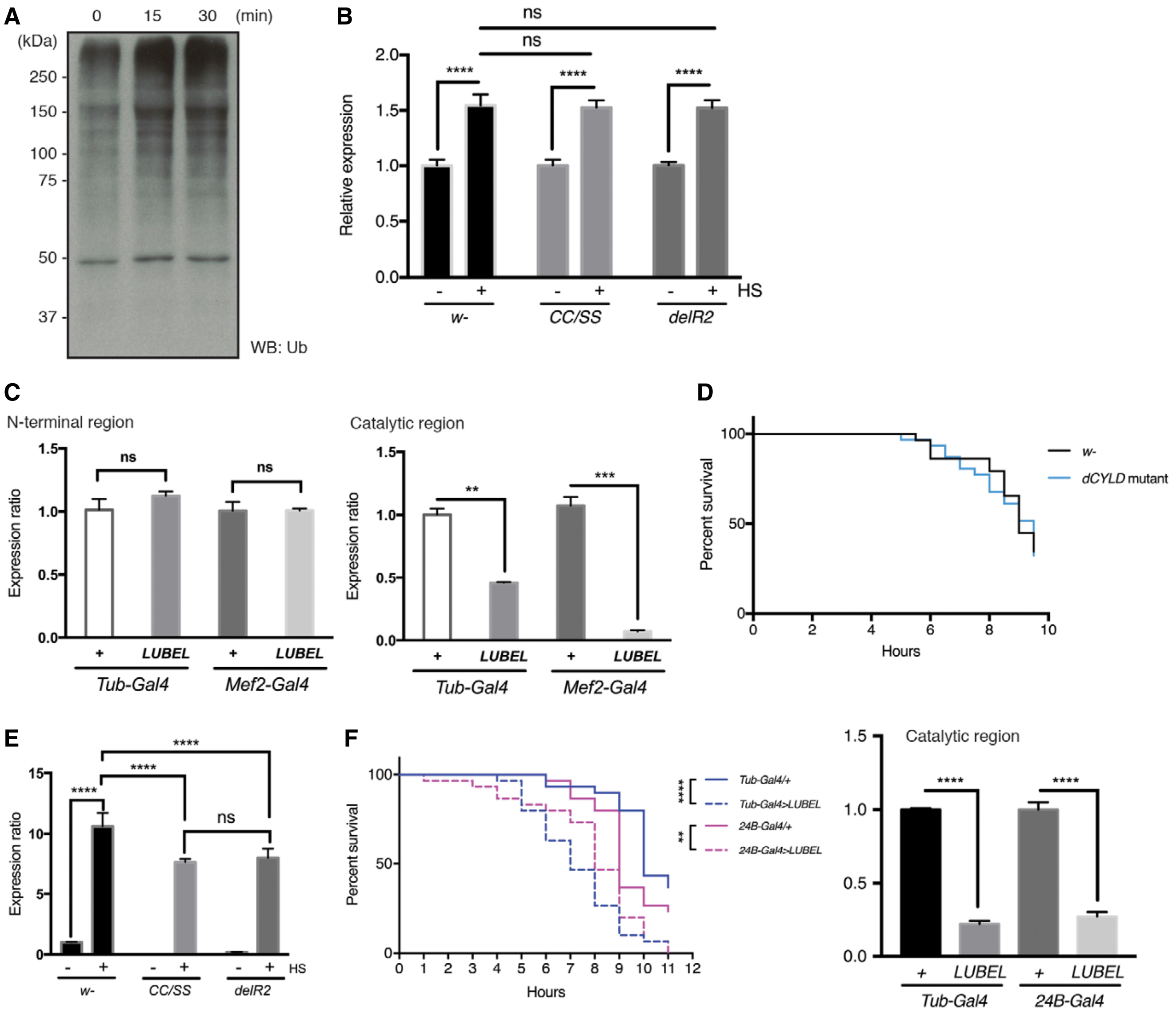

Figure EV5. Heat shock responses in ω^{-}, *dCYLD* mutant, and LUBEL knockdown (KD) flies.

A Immunoblotting for total Ub chains in heat-shocked ω^{-} flies in Fig 6A using anti-pan Ub antibody.

B mRNA expression of poly-ubiquitin gene *ubi-p63E* in LUBEL mutant flies, untreated, or heat shocked. 10 male and 10 female adult flies were heat shocked for 1 h and RNA was isolated and *Ubi-p63E* mRNA level was measured by qPCR. *Rp49* was used as reference and untreated samples were used as calibrator for each fly line to calculate the expression ratio. Data are analyzed by two-way ANOVA with multiple comparison, and presented as mean ± SD (*****P* < 0.001). Representative of three experiments is shown.

C Expression of LUBEL mRNA in heterozygous flies (*Tub-Gal4*/+ and *Mef2-Gal4*/+) and *UAS-shLUBEL* (*Tub-Gal4* > *LUBEL* and *Mef2-Gal4* > *LUBEL*) flies detected by qPCR. The primers that target N-terminal region (left panel) or catalytic region (right panel) of LUBEL were used. *Rp49* was used as reference, and GD control fly line was used as calibrator to calculate the expression ratio. Data were analyzed using unpaired *t*-test; values represent mean ± SD (***P* < 0.01, ****P* < 0.001), (*n* = 3). Representative of three independent experiments are shown.

D Survival of *dCYLD* mutant flies upon heat shock. Survival curve of heat-treated ω^{-} and *dCYLD* mutant flies is shown. 15 male and 15 female files per each fly line were used in this assay. Curve comparison tests indicated the difference is not significant. Representative data are shown from four independent experiments.

E Heat-induced mRNA expression of HSP70. ω^{-}, *CC/SS* #2, and *delR2* flies were heat treated for 30 min and mRNA HSP70 was quantified by qPCR. Rp49 was used as a reference and ω^{-} untreated sample was used as calibrator to calculate the expression ratio. Data are analyzed by two-way ANOVA with multiple comparison, and presented as mean ± SD (*****P* < 0.0001). Representative of three experiments is shown.

F Repeat of muscle-specific LUBEL KD using *B24-Gal4* driver. A heat-hock survival assay was performed as Fig 6C, using *24B-Gal4* driver line to knockdown LUBEL in the muscle. *P*-values calculated by Gehan–Breslow–Wilcoxon test: *Tub-Gal4* < 0.0001 (****), *24B-Gal4* = 0.0066 (**). knockdown efficiency for the catalytic region, analyzed as in (C), is shown on the right graph. Representative of three experiments is shown.
